# Supplementary material for: Comparative multiomics analysis of cell physiological state after culture in a basket bioreactor
Source: Sci Rep. 2022 Nov 23;12:20161. doi: 10.1038/s41598-022-24687-4 (PMC9686226; doi:10.1038/s41598-022-24687-4)
Supplement: Supplementary file 1 — Supplementary Information 1. [file 41598_2022_24687_MOESM1_ESM.zip › raw data/Proteomic raw data/6.KEGGenrichment/EG.vs.CG/ALL/pathway/src/00480.html]

map00480


- K00432, 1.11.1.9, R00274"

- Down regulated proteins

A0A0D9RRE1(Description:Glutathione peroxidase;Gene:GPX1;log2FC:-1.3844)

- K00799, K04097, K13299, K21888, 2.5.1.18, R03522"

- Up regulated proteins


A0A0D9R7G0(Description:Glutathione transferase;Gene:MGST1;log2FC:1.2430)

Close
